# Patients and generative AI: Who owns your diagnosis?

**DOI:** 10.1002/bco2.420

**Published:** 2024-10-24

**Authors:** Asher Mandel, Michael DeMeo, Ashutosh Maheshwari, Ash Tewari

**Affiliations:** ^1^ Department of Urology Mount Sinai Hospital New York NY USA

**Keywords:** artificial intelligence, intellectual property, machine learning, research and development, urology

Generative artificial intelligence (AI) chatbots, like Open AI's ChatGPT, have revolutionized the way that humans interact with machines. With a recent market capitalization of $80 billion, investors strongly believe that AI has a future role in many industries. Mounting excitement, however, is also met by cautionary discourse regarding the need for ethical shepherding of AI's rollout. Several United States Congress hearings have centred around AI with the media abuzz with its consequences. Controversies yet to be settled include how to address the use of AI in academic publishing, education and medicine, among others.^1^, ^2^, ^3^ An analysis of public perspectives on comfortability with AI in healthcare, drawn from social media content, found drastic heterogeneity.^4^ Results from a recent Pew survey suggest that higher academic level and experience with AI increases the likelihood of having confidence in AI's ability to enhance medical care.^5^ Nonetheless, natural language processing has already begun its infusion into the medical field with use cases including electrocardiogram interpretation and white blood cell count differentials.^6^

Urology is no exception in this regard—embracing the benefits of AI by exploring the utility of agents (i.e., text/voice/video chatbots) and evaluating surgical skill.^7^, ^8^ Some products have already resulted in the United States Food and Drug Administration approval, such as one that assists in localizing prostate tumour volume on magnetic resonance imaging and another that diagnoses prostate cancer on histopathology.^9^, ^10^

As AI is increasingly adopted in everyday urology practice, to improve efficiency and quality of care, it is imperative that we consider the looming ethical ramifications proactively. A recent review presented by Dr. Hung et al. has illuminated some of these challenges, stirred conversation and presented possible policy‐level solutions.^11^ Nevertheless, urologists have still yet to address several other legal and ethical challenges looming in generative AI model development. This editorial seeks to expand the scope of conversation encompassing necessary considerations for adopting AI in urology.

Three important issues to consider include the agency of patients and their data, ownership over the models themselves and the potential competition these models may add in the marketplace. Healthcare institutions are charged with being ethical stewards of patient data. This paternal identity may engender a sense of entitlement over the data, and institutions may act as though they own patient data and use that argument in negotiations; however, these data cannot be legally copyrighted. Second, healthcare systems and AI companies are competing and collaborating in this emerging space. Both may be entitled to the products they develop because while a hospital brings the patient data to the table, an AI company brings the machine learning models. Should they be 50–50 partners? What is a fair split? Finally, doctors may be contributing to the development of tools that will automate components of their jobs, which may impact the demand for their services in the marketplace. How might we consider this factor in establishing fair partnerships?

The first issue to consider is whether the patients themselves should be represented as stakeholders in the AI commercialization negotiations. Patients often consent that their data may be used for research purposes, but no one would opt out of potentially realizing profits from their data being used to bring an AI tool to market. While no cases have been litigated directly on patient data used for AI model training, there are landmark bioethical cases in genetics and tissue banking with potentially informative parallels. In 2023, the descendants of Henrietta Lacks settled a lawsuit with Thermo Fisher Scientific over the profiting of her immortalized cell lines, which had been sold in a number of commercial capacities.^12^ Regarding genetics, in Greenberg v. Miami Children's Hospital Research Institute, lawyers argued that donated tissue from patients led to the hospital's patenting of a genetic variant causing a rare disease called Canavan and commercialization of screening tests. The courts upheld the claim of unjust enrichment, stating that the patients should also share in the revenue from the royalties earned by the hospital.^13^ In these cases of tissue banking and genetic variant identification, patients have been viewed as deserving of profit sharing.

As AI models are poised to champion the next revolution in healthcare products, it is important to consider the practicality of implementing a proactive system that addresses the implications of these precedents. Offering patients compensation for their data used in AI product commercialization is a progressive idea that respects patient rights. However, most of these products are developed from retrospective research initiatives using large patient databases that are often mandated to be de‐identified by the Institutional Review Board (IRB). The process of re‐identifying these patients may jeopardize patient privacy. Additionally, the administrative task of negotiating these contracts with individual patients is a large challenge that is not currently accounted for in research budgets.

The second issue essentially hinges on how to organize a fair and just economic framework to characterize the relationships between healthcare systems and for‐profit companies who are collaborating to develop the AI models that may ultimately become commercialized clinical tools. The inputs of these models are patient data, which is stringently policed by the stipulations of the Health Insurance Portability and Accountability Act (HIPAA) and the IRB. [Correction added on 7 November 2024, after first online publication: The abbreviation, HIPPA has been corrected to HIPAA in the preceding sentence.] Companies cannot innovate without access to this data, but if the hospitals share the data they also might expect to share in future revenue. Although hospitals cannot copyright the data, they may leverage their positions as stewards of patient data to negotiate from the perspectives of pseudo‐owners.^14^ Nevertheless, negotiating these contracts can be exceedingly complex requiring significant legal and regulatory considerations that serve as stumbling blocks in the current status quo.

Let's look at a hypothetical example. A urology department wants to partner with a company to develop a tool that can augment the data gleaned from a transrectal ultrasound. They want a 360° sweep of the prostate to be recorded as a video and fed through a model that can tell the urologist the length of the prostatic urethra, the volume of the prostate, and whether there are any abnormal lesions that may be concerning for cancer. They approach the company and negotiate to reach a fair partnership and reimbursement sharing structure for the ultimate commercialization of this product. The practice has invested significant resources in the establishment and maintenance of clinical practice that has led to the creation of patient data. Therefore, they should be compensated commensurately for any tool that is built upon that foundation of data. On the other hand, the company that has developed an understanding of deep learning brings an algorithm to the table that can transform retrospective data into clinically meaningful predictions. Ultimately, the tool cannot be created without this marriage of data and algorithm.

Let's consider how the courts have approached similar cases in recent history. Thus far, the US courts have litigated fair use of data in training AI models, but only in the context of copyrighted data, which limits their applicability in healthcare where patient data cannot be copyrighted.^14^ Nonetheless, the rulings are worth exploring to shed light on the perspective of the courts in navigating these unchartered waters.

The *New York Times* is suing OpenAI for compensation since OpenAI trained its model on millions of the periodical's copyrighted articles.^15^ This lawsuit is ongoing, and many believe that the supreme court will ultimately hear this case, as similar filings are anticipated, and a generalizable ruling will be needed to guide subsequent adjudications. The core principle is called ‘fair use’, which embodies a legal defence that can permit the use of copyrighted material under certain circumstances.

Another intellectual property consideration is referred to as infringing derivative work. Namely, is the output of the model so similar to the input, that it does not actually add new value, but is merely copying? Investigating analogous cases litigated in media, academia, music and other industries, mixed rulings have been issued by the courts. Examples include Kadrey v. Meta Platforms Inc., where the plaintiff was a publishing company, and Thomson Reuters v. Ross Intelligence Inc., where the plaintiff was a legal archive. Essentially the courts have ruled that the outputs of the models are sufficiently different so as not to constitute infringing derivative work.^16^, ^17^ What the courts clarified is that using copyrighted material without permission as model inputs is not permitted, but the outputs are probably sufficiently unique to constitute being its own entity and to add new value to society.

The third issue that must be addressed is the potential disruption these technologies may induce in the demand for professional services. A pathologist trains for many years to make histopathological interpretations. The pathologist's interpretations are required as inputs for the AI model's training. If the AI model can then be used instead of the pathologist, the pathologist will lose out on potential earnings. This potential economic loss evokes hesitancy in collaboration. Therefore, maintaining ownership within the medical profession and ensuring financial benefit would possibly alleviate this hesitancy and open the floodgates of willing collaboration. Even still, this transition will be challenging given the potential resistance to change and the need for new skills training. Alternatively, adjusting the role of some physicians to act as supervisors over the AI models may be another solution. Nonetheless, automation is a wider economic phenomenon in need of address.

A final thought—all stakeholders here deserve consideration. Successful collaborations will depend on this understanding to maintain compliance and to foster investment. There would be no Napster without musicians recording music, no YouTube without content creators. There can be no chest radiography AI assistant without patients having pneumonia and radiologists writing their impressions. It is in all our interests that these tools be trained on as much data as possible to ensure they are robust and generalizable across communities. Let's take a pause and realize that we have all the ingredients to create these tools. If we can proceed with a spirit of generosity and be mindful of everyone's contributions, there will be no stopping the great promise of clinical revolution made possible by generative AI.

## CONFLICT OF INTEREST STATEMENT

Dr. Tewari discloses holding non‐financial leadership positions in The Kalyani Prostate Cancer Institute, The Global Prostate Cancer Foundation, Roivant, PathomIQ, and Intuitive Surgical. He has served as a site‐PI on pharma/industry sponsored clinical trials from Kite Pharma Inc., Lumicell Inx., Dendron Pharmaceuticals, LLC, Oncovir Inc., Blue Earth Diagnostics Ltd., RhoVac ApS., Bayer HealthCare Pharmaceuticals Inc., Janssen Research and Development, LLC. Dr. Tewari has served as an unpaid consultant to Roivant Biosciences and advisor to Promaxo. He owns equity in Promaxo.

Asher Mandel has nothing to disclose. Michael DeMeo has nothing to disclose. Ashutosh Maheshwari has nothing to disclose.
